# Point Prevalence Surveys of Antimicrobial Use among Hospitalized Children in Six Hospitals in India in 2016

**DOI:** 10.3390/antibiotics6030019

**Published:** 2017-09-13

**Authors:** Sumanth Gandra, Sanjeev K. Singh, Dasaratha R. Jinka, Ravishankar Kanithi, Ashok K. Chikkappa, Anita Sharma, Dhanya Dharmapalan, Anil Kumar Vasudevan, Onkaraiah Tunga, Akhila Akula, Garima Garg, Yingfen Hsia, Srinivas Murki, Gerardo Alvarez-Uria, Mike Sharland, Ramanan Laxminarayan

**Affiliations:** 1 Center for Disease Dynamics, Economics & Policy, New Delhi 110020, India; ramanan@cddep.org; 2 Department of Infection Control & Microbiology, Amrita Institute of Medical Sciences, Amrita University, Ponekkara, Kochi 682041, India; sanjeevksingh@aims.amrita.edu (S.K.S.); vanilkumar@aims.amrita.edu (A.K.V.); 3 Department of Infectious Diseases & Department of Paediatrics, Rural Development Trust Hospital, Bathalapalli 515661, India; jdashrath86@gmail.com (D.R.J.); gerardouria@gmail.com (G.A.-U.); 4 Department of Paediatrics, Sowmya Children’s Hospital, Hyderabad 500038, India; ravineonatologist@gmail.com (R.K.); akhi0602@gmail.com (A.A.); 5 Department of Paediatrics, Rural Development Trust Hospital, Kalyanadurgam 515761, India; ashokkld1971@yahoo.com (A.K.C.); onkaraiahtunga@gmail.com (O.T.); 6 Department of Microbiology & Department of Paediatric Intensive Care Unit, Fortis Hospital, Mohali 160062, India; anita.sharma@fortishealthcare.com (A.S.); Garima.garg@fortishealthcare.com (G.G.); 7 Department of Paediatrics, Dr Yewale’s Multispeciality Hospital for Children, Navi Mumbai 400703, India; drdhanyaroshan@gmail.com; 8 Paediatric Infectious Diseases Research Group, Institute of Infection and Immunity, St. Georges University, London SW17 0RE, UK; yhsia@sgul.ac.uk (Y.H.); Mike.Sharland@stgeorges.nhs.uk (M.S.); 9 Department of Neonatology, Fernandez Hospital, Hyderabad 500029, India; srinivasmurki2001@gmail.com; 10 Princeton Environmental Institute, Princeton University, Princeton, NJ 08544, USA

**Keywords:** point prevalence survey, antimicrobial use, children, hospital, India

## Abstract

The prevalence of antimicrobial resistance in India is among the highest in the world. Antimicrobial use in inpatient settings is an important driver of resistance, but is poorly characterized, particularly in hospitalized children. In this study, conducted as part of the Global Antimicrobial Resistance, Prescribing, and Efficacy in Neonates and Children (GARPEC) project, we examined the prevalence of and indications of antimicrobial use, as well as antimicrobial agents used among hospitalized children by conducting four point prevalence surveys in six hospitals between February 2016 and February 2017. A total of 681 children were hospitalized in six hospitals across all survey days, and 419 (61.5%) were prescribed one or more antimicrobials (antibacterials, antivirals, antifungals). Antibacterial agents accounted for 90.8% (547/602) of the total antimicrobial prescriptions, of which third-generation cephalosporins (3GCs) accounted for 38.9% (213/547) and penicillin plus enzyme inhibitor combinations accounted for 14.4% (79/547). Lower respiratory tract infection (LRTI) was the most common indication for prescribing antimicrobials (149 prescriptions; 24.8%). Although national guidelines recommend the use of penicillin and combinations as first-line agents for LRTI, 3GCs were the most commonly prescribed antibacterial agents (55/149 LRTI prescriptions; 36.9%). In conclusion, 61.5% of hospitalized children were on at least one antimicrobial agent, with excessive use of 3GCs. Hence there is an opportunity to limit their inappropriate use.

## 1. Introduction

Antimicrobial resistance is rising across the globe, with prevalence in India reported as being among the highest [1,2]. A recent study reported that resistance to last-resort antimicrobials increased between 2008 and 2014 [3]. In 2014, 57% of *Klebsiella pneumoniae* and 10% of *Escherichia coli* blood culture isolates were observed to be carbapenem resistant. The high proportion of bacterial infections due to extended spectrum beta-lactamase (ESBL)-producing organisms is one reason for the high consumption of carbapenems in India. Among 51 countries for which antimicrobial resistance surveillance data were available in 2014, India had the highest proportion of third-generation cephalosporin-resistant *E. coli* (83%), an indirect marker for ESBL production [4].

Antimicrobial selection pressure is a primary driver of resistance development [5], and there is an urgent need to reduce antimicrobial overuse and misuse. Surveillance of antimicrobial use in hospitals can provide an insight into patterns of antimicrobial use, help highlight differences in prescribing practices among hospitals, and identify opportunities for improvement. Information on antimicrobial use from point prevalence surveys (PPSs) could be used to design, implement, and assess the effects of antimicrobial policies [6]. To date, only one multicenter (>2 hospitals) study describing antimicrobial use among hospitalized children has been published in India [7]. However, this study did not collect information on the total number of children admitted to various wards and thus could not estimate the rate of antimicrobial use per patient. In this study, we examined the prevalence of and indications of antimicrobial use, as well as the antimicrobial agents used among hospitalized children, by conducting four PPSs in six hospitals in India.

## 2. Methods

As part of the Global Antimicrobial Resistance, Prescribing, and Efficacy in Neonates and Children (GARPEC) study, the participating hospitals were asked to conduct a one-day cross-sectional hospital based PPSs in all pediatric and neonatal wards four times between 1 February 2016 and 28 February 2017. The Antibiotic Resistance and Prescribing in European Children (ARPEC) project PPS methodology was utilized for this study [8]. Four single-day PPSs on antimicrobial use were conducted between 1 February 2016 and 28 February 2017. The first PPS was conducted between 1 February and 31 March 2016; the second between 1 May and 30 June 2016; the third between 1 September and 31 October 2016; and the fourth between 1 December 2016 and 28 February 2017. Four PPSs were conducted to increase the precision of measurement of antimicrobial use and to examine the variation of antimicrobial use at different time points.

Each hospital needed to register providing the name, geographic location and type of hospital (primary, secondary and tertiary level and teaching vs. nonteaching hospital). Hospitals were asked to conduct the survey only on a weekday during the designated months of each round of PPS. All neonates and pediatric hospitalized patients younger than 18 years of age, present in the ward at 8:00 a.m., were included in the survey. Detailed data were recorded only for patients with an active antimicrobial prescription at 8 am on the day of survey.

At the time of initiation of the study on 1 February 2016, five hospitals were enrolled into the study. Three additional hospitals were enrolled by 1 May 2016 and did not participate in the first round of PPS. Among the eight hospitals, two were rural general trust hospitals, three were stand-alone private children’s hospitals, two were private tertiary care hospitals and one was a private mother and child care center with inborn neonatal services. One tertiary care hospital had teaching services in pediatrics and neonatal departments. Two stand-alone pediatric hospitals and the mother and child care center had teaching services in neonatal departments. PPSs were conducted in the neonatal intensive care units and neonatal wards of all eight hospitals and the pediatric units (including pediatric intensive care units) of only six hospitals (one hospital had only neonatal services and the other hospital restricted the study to neonatal units). In this study, we examined the antimicrobial use patterns among the pediatric units of six hospitals. Antimicrobial use in neonatal intensive care units and general neonatal wards were not examined in this study.

Hospital, department, and de-identified patient data were collected using a standardized web-based electronic data entry form on the Research Electronic Data Capture (REDCap)^®^ developed for the GARPEC project. For children receiving antimicrobials (antibiotics, antifungal and antivirals, antiparasital agents), data were collected on patient sex, age, weight, ventilation status, comorbid conditions, number of antimicrobials, antimicrobial name, dose per administration, dose units, number of doses each day, route of administration, reason for treatment, treatment indication (community versus healthcare associated) or prophylaxis, and whether treatment was empirical or targeted. We included all diagnoses for which antimicrobials were prescribed even if there was more than one diagnosis. Ethics approval was received for all participating hospitals from their respective institutional human research ethics committees.

Categorical variables were expressed as percentages, and the chi-squared test or Fisher’s exact test was used, as appropriate, for comparisons. Statistical significance was defined as *p*  < 0.05. The statistical analyses were performed using STATA 12.1 (StataCorp, College Station, TX, USA).

## 3. Results

At the six participating hospitals, the total number of beds for all four survey days ranged from 24 to 517, and the bed occupancy ranged from 15.1% to 79.8% (Table 1).

A total of 681 children were hospitalized in six hospitals across all survey days, and 419 (61.5%) were prescribed one or more antimicrobials. The percentage of children on antimicrobials in the six hospitals ranged from 55.1% to 92.9% (Table 1). One antimicrobial was prescribed to 291 patients, two antimicrobials were prescribed to 85 patients, and three or more were prescribed to 43 patients. The percentages of patients on antimicrobials for the four PPSs were 61.3% (73/119), 59.4% (104/175), 63.6% (133/209), and 61.2% (109/178), respectively.

Of the 419 children receiving antimicrobials, 147 (35.1%) were less than one year old, and 248 (59.2%) were male (Table 2).

Only one hospital (Hospital D) had two dedicated surgical wards. The remaining five hospitals had general pediatric wards, where both medical and surgical patients were admitted. Pediatric intensive care units were present in five hospitals, of which two had dedicated surgical intensive care units. General pediatric wards accounted for 78.5% (329) of children on antimicrobials, and intensive care units accounted for the remaining 21.5% (90) (Table 2). The percentage of patients on antimicrobials was significantly higher in intensive care units than in general pediatric wards (73.4% vs. 59.0%; *p* = 0.003). The average number of antimicrobials per patient was higher in intensive care units (mean 1.5; range 1–4) than in general pediatric wards (mean 1.4; range 1–5). In 419 patients with an antimicrobial prescription, 343 (81.8%) were prescribed for treatment of active infection and 76 (18.2%) for prophylaxis. Lower respiratory tract infections (LRTIs) (117, 27.9%), sepsis (66, 15.7%), and prophylaxis for surgical disease (49, 11.7%) were the three most common reasons for prescribing antimicrobials (Table 2).

Of the 602 total antimicrobial prescriptions, 313 (52%) were for community-acquired infections (CAIs), 55 (9.1%) were for healthcare associated infections (HAI), and 116 (19.3%) were for unknown indications (CAI or HAI) (Table 2). Of the 602 prescriptions, 118 (19.6%) were for medical and surgical prophylaxis. Among the 313 prescriptions for CAIs, 283 (90.4%) were empiric, whereas 35 (63.6%) of 55 prescriptions for HAIs were empiric. Of the 313 antimicrobial prescriptions for CAIs, the majority were prescribed for LRTI (133 prescriptions; 42.5%) and sepsis (54 prescriptions; 17.3%).

Antibacterial agents accounted for 90.8% (547 of 602) of total antimicrobial prescriptions. Of the 547 antibacterial prescriptions, the three most common classes of antibiotics prescribed were third-generation cephalosporins (213 of 547 prescriptions; 38.9%), penicillin plus enzyme inhibitor combinations (78 of 547 prescriptions; 14.3%), and aminoglycosides (57 of 547 prescriptions; 10.4%) (Table 3).

The top three classes of antibacterial agents and their order were similar in each of the four PPSs. Carbapenems and fluoroquinolones accounted for less than 5% each of total antibacterial prescriptions, respectively (Table 3).

**Figure 1:**
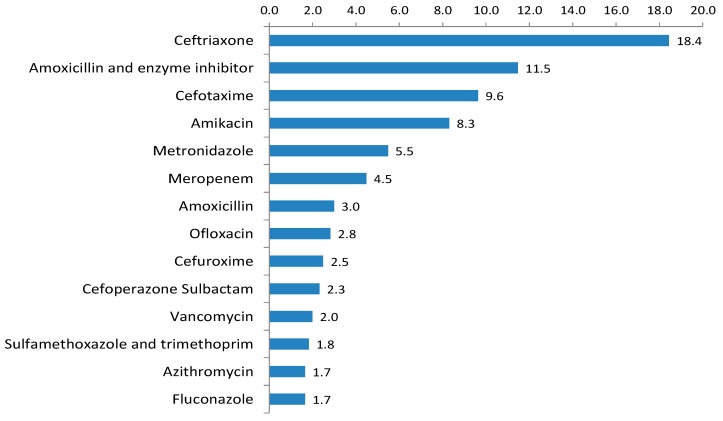
Prescribed antimicrobials among hospitalized children, ranked by overall drug utilization 75% (DU75%).

Of the prescriptions for carbapenems, 55.5% (15/27) were prescribed in intensive care units. The top three reasons for prescribing carbapenems were sepsis (4 HAI and 1 CAI), LRTI (3 CAI and 1 HAI), and central nervous system infections (2 CAI and 1 unknown). Overall, the three most commonly prescribed antimicrobials were ceftriaxone (111 of 602 prescriptions; 18.4%), amoxicillin-clavulanic acid (69 of 602 prescriptions; 11.5%), and cefotaxime (58 of 602 prescriptions; 9.6%) (Figure 1).

The five diagnoses associated with the highest rates of antimicrobial prescribing were LRTI (149 of 602 prescriptions; 24.8%), sepsis (90 of 602 prescriptions; 15%), prophylaxis for surgical disease (77 of 602 prescriptions; 12.8%), treatment of surgical disease (64 of 602 prescriptions; 10.6%), and prophylaxis for medical problems (41 of 602 prescriptions; 6.8%) (Table 2). The two most commonly prescribed antibacterial classes for LRTI were third-generation cephalosporins (55 of 149 prescriptions; 36.9%) and penicillin plus enzyme inhibitor combinations (46 of 149 prescriptions; 30.9%). For LRTI, third-generation cephalosporins were mainly used as monotherapy (38/55) and in combination in 17 other cases, mostly with macrolides (5/17). The two most common antibacterial agents prescribed for sepsis were the third-generation cephalosporins (40 of 90 prescriptions; 44.4%) and amikacin (11 of 90 prescriptions; 12.2%). For sepsis cases, third-generation cephalosporins were mainly used as monotherapy (26/40) and in combination in 14 other cases, mostly with amikacin (8/14).

In cases of prophylaxis for surgical disease, the two most commonly prescribed antibacterial classes were third-generation cephalosporins (29 of 64 prescriptions; 37.7%) and aminoglycosides (16 of 64 prescriptions; 25%). Third-generation cephalosporins were used as monotherapy in 9 cases and in combination in 20 other cases, mostly with aminoglycosides (8/20) and with both aminoglycosides and metronidazole (4/20). Third-generation cephalosporins were the most commonly used antibacterial agents (14/41) used for medical prophylaxis. They were used as monotherapy in six patients in combination mainly with aminoglycosides in eight patients (4/8).

## 4. Discussion

To the best of our knowledge, this is the first published multicenter study to estimate the prevalence of antimicrobial use among hospitalized children in India. The percentage of hospitalized children who received at least one antimicrobial agent was 61.5% in this study. In the global Antibiotic Resistance and Prescribing in European Children (ARPEC) PPS study conducted in 2012, involving 226 hospitals from 41 countries, the overall percentage of hospitalized children on antimicrobials was 42.5%, which was much lower than the 61.5% in our study [8]. The percentage of patients on antimicrobials in our study was higher than reported in Turkey (54.6%) [9], Italy (47%) [10], Australia (46%) [11], the United Kingdom (40.9%) [12], Latvia (39%) [13], and the United States (33%) [14]. However, it was lower than in Iran (66.6%) [15] and China (78.2%) [16]. Consistent with previous studies, we observed that the majority of the antimicrobial usage was for therapeutic purposes rather than prophylaxis [8,10,11,12,15].

LRTI was the most common indication for prescribing antimicrobials, a finding consistent with PPSs among hospitalized children in several other countries [10,11,12,13]. Third-generation cephalosporins were the most commonly prescribed antibiotics for LRTI in this study. Similar findings were reported by another Indian study, which reviewed antibiotic prescription practices among hospitalized children in two private hospitals in central India [17]. However, the Indian National Center for Disease Control and Prevention (NCDC) guidelines [18] and the INDIACLEN task force guidelines for pneumonia [19] recommend ampicillin or ampicillin plus gentamicin or amoxicillin-clavulanic acid as first-line therapy for LRTI among hospitalized children older than two months. Third-generation cephalosporins are recommended in hospitalized children only when they deteriorate on first-line agents.

Consistent with our findings, third-generation cephalosporins were the most commonly prescribed antimicrobials in Eastern Europe (35.7%) and Asia (28.6%) in the global ARPEC study [8], as well as in Turkey (18.4%) [9], Italy (20%) [10], Latvia (28%) [13], and Iran (43.5%) [15]. However, in Australia and the United Kingdom, penicillin plus enzyme inhibitor combinations were the most commonly prescribed antimicrobials [11,12]. Carbapenem prescription in this study was lower than in Turkey (12.7%) [9], Italy (6%) [10], and Iran (5.2%) [15], but higher than in Australia (3.2%) [11] and Latvia (0.5%) [13]. However, it is possible that carbapenem consumption has been underestimated because of the lack of representation of tertiary care centers with large numbers of intensive care unit beds in this study.

The study has several strengths. First, the repeated PPSs in the six hospitals increased the robustness of our estimates of antimicrobial prescription among hospitalized children. Second, the six participating hospitals represented diverse settings that are commonly seen in India. Two hospitals were small stand-alone children hospitals in urban areas, two were part of a rural general hospital, and two were part of large tertiary care referral centers. Thus we were able to capture antimicrobial prescribing practices in different hospital settings. Third, we examined for the variation of antimicrobial use at different times of the year. Interestingly, the percentages of children on antimicrobials and the antimicrobial prescription patterns in each of the four PPSs were similar when compared with combined data from all four PPSs. We did not observe any temporal variation in antimicrobial prescribing.

The study also has several limitations, however. First, we did not collect data on the duration of therapy, nor the microbiology and antimicrobial susceptibility results, which could help indicate the appropriateness of antimicrobial prescribing; Second, although we included hospitals with different characteristics, we did not include large academic centers and hospitals from all regions of India. This would require a much larger study. Thus, our results may not be generalizable to all healthcare settings and geographic regions of the country. Similarly, as all hospitals included in the study were from the private sector, the results may not be generalizable to public sector hospitals. The choice of antibiotics may differ in public hospitals as the national and state drug policies often define the types of antibiotics procured and prescribed in the public sector hospitals. The public sector hospitals are also obliged to follow national prescribing guidelines; Third, five out of six hospitals did not have dedicated surgical wards or other specialized units such as hematology-oncology or cardiology, which limited our ability to study variation of antimicrobial use across different hospital units; Fourth, with Hospital D having most number of beds and patients in the study, the results could have been biased affecting the representativeness of the data; Fifth, the date of the survey was chosen as per the convenience of the site principal investigator within the specified months. However, we have not taken additional steps to minimize the Hawthorne effect. As the physicians knew that their antimicrobial prescriptions were being studied, the results might have been affected. Sixth, 79 (19%) of the prescriptions had unknown indications (where it was not known whether the infection was community acquired or healthcare associated), however, we have not taken additional efforts to identify the indications.

Our study identified an opportunity to improve antimicrobial use for LRTI among hospitalized children. The current guidelines recommend the use of third-generation cephalosporins only when there is deterioration in the effectiveness of first-line agents. However, in our study, the majority of hospitalized children with LRTI were on third-generation cephalosporins. A recent study assessing the antibiotic susceptibility of the major bacterial pathogens isolated from community-acquired pneumonia among both adults and children in India indicated high susceptibility to first-line agents (ampicillin and amoxicillin-clavulanic acid) [20]. In this study, 91.8% of *Streptococcus pneumoniae* isolates were susceptible to amoxicillin. Similarly, 91.1% of *Haemophilus influenzae* isolates were susceptible to ampicillin, and 97% of the *H. influenzae* isolates were susceptible to amoxicillin-clavulanic acid [20]. This study reinforces that third-generation cephalosporins can be avoided as first-line therapy for LRTI. We also observed that third-generation cephalosporins were the most commonly used antibiotics for surgical prophylaxis. However, the published international guidelines recommend use of first- and second-generation cephalosporins for surgical prophylaxis instead of third-generation cephalosporins [21].

Reviewing national antimicrobial resistance surveillance data obtained both from adults and children, in 2014, 83% of the *E. coli* isolates were resistant to third-generation cephalosporins in India, which was much higher than in Australia (9%), the United Kingdom (11%), Argentina (14%), the United States (16%), South Africa (19%), and China (62%) [22,23]. Use of third-generation cephalosporins is associated with increased the risk of colonization with ESBL-producing bacteria [24]. Third-generation cephalosporin exposure in children could lead to colonization at a very young age, which could facilitate the spread of ESBL-producing bacteria to other family members, leading to a further increase in ESBL Enterobacteriaceae infections and subsequently the consumption of carbapenems in India. Our findings indicate the need for increased compliance with NCDC and INDIACLEN treatment guidelines for the management of LRTI in children.

In conclusion, 61.5% of the hospitalized children were on at least one antimicrobial agent. We observed an excessive use of third-generation cephalosporins for LRTI. There is an opportunity to limit the use of third-generation cephalosporins by following the recommended national treatment guidelines for management of LRTI.

## Figures and Tables

**Table 1:** Characteristics, bed occupancy, and antimicrobial prescription in six hospitals in India in 2016.

Hospital ID	Hospital Characteristics	Total Beds	Total Patients (N)	Bed Occupancy (%)	Intensive Care Beds (Yes/No)	Patients on Antimicrobials (N)	Patients on Antimicrobials (%)
A *	Rural general hospital	149	92	61.7	Y	51	55.4
B	Stand-alone pediatric	112	79	70.5	Y	57	72.2
C *	Rural general hospital	119	95	79.8	N	76	80.0
D ^#^	Tertiary care hospital	517	385	74.5	Y	212	55.1
E *	Tertiary care hospital	24	14	58.3	Y	13	92.9
F **	Stand-alone pediatric	106	16	15.1	Y	10	62.5
All		1027	681	66.3		419	61.5

Note: Hospitals A, B, and F have only medical intensive care units; Hospitals D and E have surgical intensive care beds available in addition to medical. * Did not participate in the first point prevalence survey; ** Did not participate in the second point prevalence survey; ^#^ Has teaching services in pediatric departments.

**Table 2:** Percentages of hospitalized children on antimicrobials in six hospitals in India in 2016.

Characteristic	Number of Children (*N* = 419)	Percentage of Children	Number of Prescriptions (*N* = 602)	Percentage of Prescriptions
Underlying comorbid conditions	256	61.1	359	59.6
No underlying disease	163	38.9	243	40.4
**Age category**				
Age < 1	147	35.1	197	32.7
Age 1–6	173	41.2	255	43.2
Age 7–12	79	18.8	118	19.6
Age > 12	20	4.8	32	5.5
**Gender**				
Male	248	59.2	354	58.6
**Ward activity**				
Intensive care units	90	21.5	138	22.9
General wards	329	78.5	464	77.1
Diagnosis *				
Lower respiratory tract infection (LRTI)	117	27.9	149	24.8
Sepsis	67	15.9	90	15.0
Prophylaxis for surgical disease	50	11.9	77	12.8
Treatment for surgical disease	34	8.1	64	10.6
Prophylaxis for medical problems	29	6.9	41	6.6
Other	80	19.0	116	19.3
Upper respiratory infections (URTI)	17	4.1	23	3.8
Urinary tract infections (UTI)	18	4.3	22	3.7
GI tract infections	19	4.5	21	3.5
Indication *				
Community-acquired infection (CAI)	233	55.6	313	52.0
Healthcare-associated infection (HAI)	40	9.5	55	9.1
Unknown	79	18.9	116	19.3
Prophylaxis (medical and surgical)	79	18.9	118	19.6

* Total can be more than 100% as one patient can have more than one diagnosis.

**Table 3:** Antimicrobial prescriptions for all indications among hospitalized children in six hospitals in India in 2016.

	All	Hospital A	Hospital B	Hospital C	Hospital D	Hospital E	Hospital F
Children on ≥1 antimicrobials	432	93	108	95	388	14	14
Total number of prescriptions	**602**	**77**	**90**	**91**	**306**	**26**	**12**
Third-generation cephalosporins	**213**	25	44	16	108	12	8
Penicillin + enzyme inhibitors	**78**	13	3	44	16	1	1
Others *	**73**	13	16	19	22	3	0
Aminoglycosides	**57**	11	4	1	31	10	0
Penicillins	**35**	4	1	0	29	0	1
Metronidazole	**33**	0	2	2	29	0	0
Carbapenems	**27**	2	3	1	21	0	0
Fluoroquinolones	**20**	1	6	0	13	0	0
First/second-generation cephalosporins	**18**	0	0	0	18	0	0
Macrolides	**16**	1	6	2	6	0	1
Glycopeptides	**14**	3	3	0	8	0	0
Trimethoprim/sulfa	**12**	3	1	4	4	0	0
Tetracycline	**6**	1	1	2	1	0	1

* Others included chloramphenicol, clindamycin, linezolid, doxycycline, tigecycline, colistin, and antituberculosis, antifungal, antiviral, and antimalarial agents.
